# PPARγ Agonists Promote Oligodendrocyte Differentiation of Neural Stem Cells by Modulating Stemness and Differentiation Genes

**DOI:** 10.1371/journal.pone.0050500

**Published:** 2012-11-21

**Authors:** Saravanan Kanakasabai, Ecaterina Pestereva, Wanida Chearwae, Sushil K. Gupta, Saif Ansari, John J. Bright

**Affiliations:** 1 Neuroscience Research Laboratory, Methodist Research Institute, Indiana University Health, Indianapolis, Indiana, United States of America; 2 Department of Medicine, Indiana University School of Medicine, Indianapolis, Indiana, United States of America; Federal University of Rio de Janeiro, Brazil

## Abstract

Neural stem cells (NSCs) are a small population of resident cells that can grow, migrate and differentiate into neuro-glial cells in the central nervous system (CNS). Peroxisome proliferator-activated receptor gamma (PPARγ) is a nuclear receptor transcription factor that regulates cell growth and differentiation. In this study we analyzed the influence of PPARγ agonists on neural stem cell growth and differentiation in culture. We found that in vitro culture of mouse NSCs in neurobasal medium with B27 in the presence of epidermal growth factor (EGF) and basic fibroblast growth factor (bFGF) induced their growth and expansion as neurospheres. Addition of all-trans retinoic acid (ATRA) and PPARγ agonist ciglitazone or 15-Deoxy-Δ^12,14^-Prostaglandin J_2_ (15d-PGJ2) resulted in a dose-dependent inhibition of cell viability and proliferation of NSCs in culture. Interestingly, NSCs cultured with PPARγ agonists, but not ATRA, showed significant increase in oligodendrocyte precursor-specific O4 and NG2 reactivity with a reduction in NSC marker nestin, in 3–7 days. In vitro treatment with PPARγ agonists and ATRA also induced modest increase in the expression of neuronal β-III tubulin and astrocyte-specific GFAP in NSCs in 3–7 days. Further analyses showed that PPARγ agonists and ATRA induced significant alterations in the expression of many stemness and differentiation genes associated with neuro-glial differentiation in NSCs. These findings highlight the influence of PPARγ agonists in promoting neuro-glial differentiation of NSCs and its significance in the treatment of neurodegenerative diseases.

## Introduction

The central nervous system (CNS) was thought to be a terminally differentiated organ. This was partly because the majority of cells in adult mammalian brain emerge at prenatal period which has limited ability to grow and replace lost cells or restore function. During embryonic development these cells originate from neural progenitor cells (NPCs) [1]–[3]. But recent studies have localized a small population of resident neural stem cells (NSCs) in the subventricular zone of adult brain [4]. It is well recognized that NSCs can proliferate, migrate and differentiate into neurons and glia in normal brain [5]–[8]. Under optimum conditions, NSCs grow and differentiate into neuro-glial cells in culture [9], [10]. Adoptively transferred NSCs migrate, grow and differentiate into neuro-glial cells in the brain of experimental models [11], suggesting their use in the treatment of human neurodegenerative diseases such as multiple sclerosis (MS), Alzheimer's disease (AD), Parkinson's disease (PD), spinal cord injury, trauma, and stroke [12]–[15]. Unfortunately, the behavior of cells in normal brain or in tissue culture does not adequately predict how these cells will behave in the CNS of patients with neurodegenerative diseases. This is more significant in compromised CNS niche with neuroinflammation where multiple factors converge on to influence the normal physiology. Thus an effective therapy for neurodegenerative diseases hinges on novel strategies to improve the ability of NSCs to thrive, integrate, and function in a physiologically meaningful manner without causing adverse side effects.

Peroxisome proliferator-activated receptors (PPAR) are a family of ligand-dependent nuclear receptor transcriptional factors that regulate lipid metabolism and glucose homeostasis [16]–[18]. PPARα, PPARγ and PPARδ are three known subtypes of the PPAR family [19]. Several fatty acids, leukotrienes and 15-Deoxy-Δ^12,14^-Prostaglandin J_2_ (15d-PGJ_2_) function as natural ligand for PPARγ [20]–[23]. Thiazolidinediones (TZDs) such as ciglitazone, troglitazone, pioglitazone, and rosiglitazone function as high affinity synthetic agonists for PPARγ [24]–[27]. Upon activation with specific ligands, PPARγ forms heterodimer complex with retinoid X receptor (RXR) and mediates target gene expression [19]. Interestingly, earlier studies have demonstrated that PPARγ is a potent regulator of inflammation [21], [28] and in vivo treatment with PPARγ agonists reduces clinical symptoms of MS, Alzheimer's disease, spinal cord injury, and stroke in animal models [29]–[31]. TZD compounds have been shown to inhibit microglial activation [32], [33] and brain injury [34]–[38]. We have demonstrated earlier that PPARγ agonists ameliorate experimental allergic encephalomyelitis (EAE) model of MS by blocking inflammatory signaling networks [39], suggesting a physiological role for the PPARγ in the regulation of inflammation and CNS repair in neurodegenerative diseases.

However, recent studies examining the influence of PPARγ agonists on cultured NSCs generated conflicting results. While many reports showed reduced growth with increased differentiation, others demonstrated increased growth with reduced differentiation of NSCs in culture [40]–[42]. In this study we examined the mechanisms by which PPARγ agonists regulate growth and differentiation of NSCs in culture. Our results demonstrate that PPARγ agonists promote oligodendrocyte differentiation of mouse NSCs by modulating the expression of stemness and differentiation genes, suggesting its use in the treatment of demyelinating diseases.

## Results

### PPARγ agonists inhibit proliferation of NSCs

**Figure 1:**
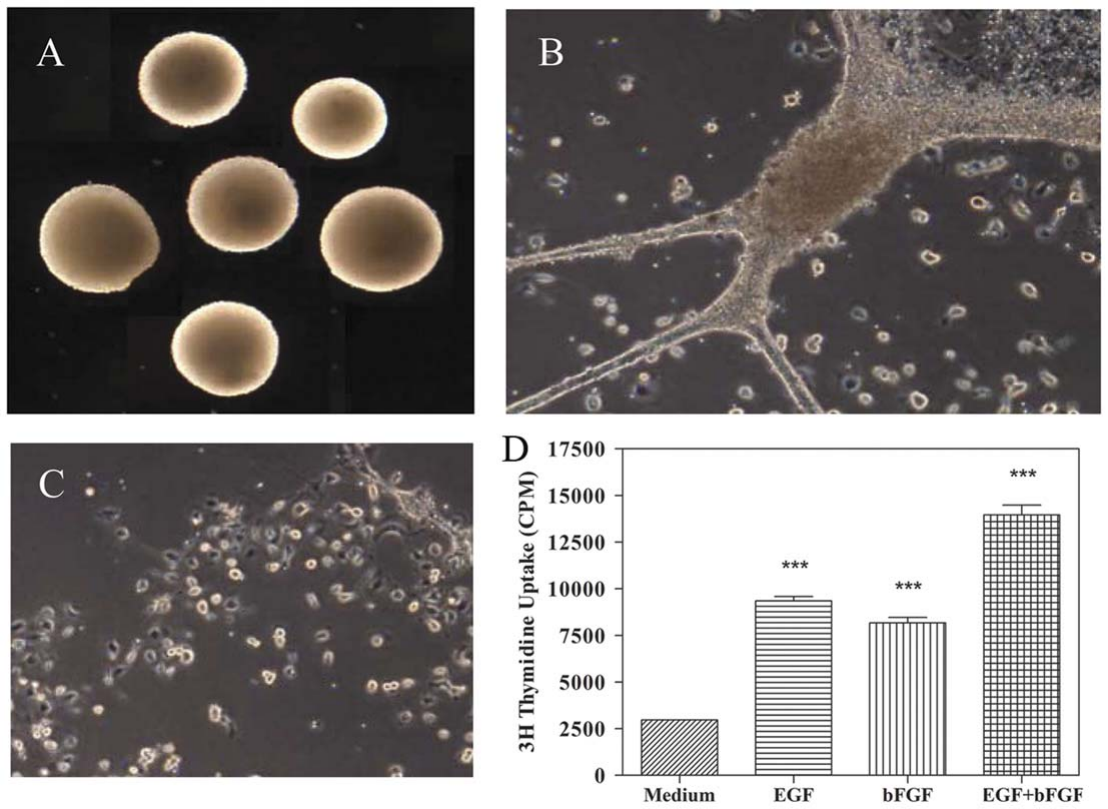
EGF+bFGF induce expansion of NSCs as neurospheres in culture. Brain cells isolated from newborn mice were cultured in 12 well tissue culture plates in NBM+B27 with EGF+bFGF. Neurospheres formed in 7–10 days were photographed under microscope (100×) (A). Neurospheres attach and spread after 10 days in culture (200×) (B). NSCs dissociated from 10 day old neurospheres (200×) (C) were cultured in 96 well tissue culture plates (1×10^4^/0.2 ml/well) and the proliferation was measured by ^3^H thymidine uptake assay (D). Values are means of triplicates±SD and the p values are expressed as *(p<0.05), **(p<0.01) and ***(p<0.001). The figure is a representative of three independent experiments.

To study the effect of PPARγ agonists on NSCs, we first established conditions to grow and expand mouse NSCs in culture. As shown in Figure 1, in vitro culture of brain cells from newborn mice in NBM with EGF+bFGF induced neurosphere formation in 3 to 5 days that increased in size by 10 days (Fig 1A, B). The NSCs dissociated from neurospheres (Fig. 1C) showed proliferation in NBM and that increased significantly following addition of EGF and bFGF alone or in combination (Fig. 1D). ^3^H Thymidine uptake assay showed that NSCs cultured in medium displayed a background count of 2970 cpm that increased to 8168±297 and 9356±223 cpm following the addition of 10 ng/ml bFGF and EGF, respectively (Fig. 1D). Moreover, addition of EGF and bFGF in combination resulted in further increase in proliferation reaching 13960±520 cpm, suggesting a potentiating effect for these growth factors on NSCs in culture.

**Figure 2:**
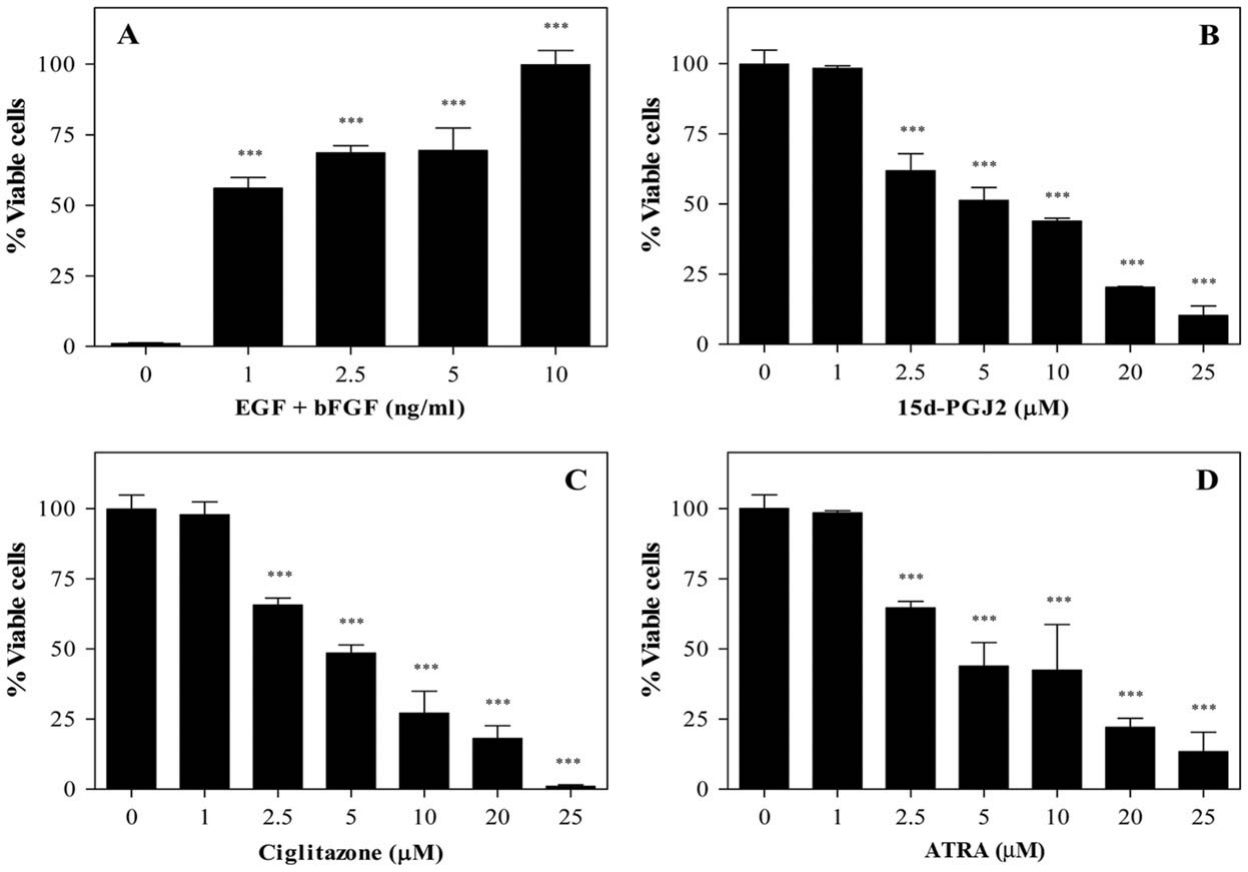
Inhibition of NSC proliferation by PPARγ agonists. NSCs dissociated from 7–10 day old neurospheres were cultured in 96 well tissue culture plates (1×10^4^/0.2 ml/well) in NBM+B27 with different doses of EGF+bFGF (A) or 10 ng/ml EGF+bFGF in the presence of different doses of 15d-PGJ_2_ (B), ciglitazone (C) and ATRA (D). The cell proliferation/viability was measured by WST-1 assay. The values are means of triplicates±SD and the p values are expressed as *(p<0.05), **(p<0.01), and ***(p<0.001). The figure is a representative of three independent experiments.

We then examined the effect of PPARγ agonists on the proliferation of NSCs in culture. As shown in Figure 2, in vitro culture of NSCs in the presence of EGF+bFGF showed a dose-dependent increase in proliferation or viable cell count as determined by WST-1 assay. Viable NSCs in the absence of growth factor was 3% that increased to 56, 68, 69 and 100 percent by the addition of 1, 2.5, 5 and 10 ng/ml EGF+bFGF, respectively in culture (Fig. 2A). Interestingly, addition of PPARγ agonists resulted in a dose-dependent decrease in viable cell count in culture (Fig. 2B, C). While NSCs cultured in NBM with EGF+bFGF in the absence of 15d-PGJ2 showed 100% viability, which decreased to 98, 62, 51, 44, 20, and 10 percent following addition of 1, 2.5, 5, 10, 20 and 25 µM 15d-PGJ2, respectively (Fig. 2B). Similarly, NSCs cultured in the absence of ciglitazone showed 100% viability, which decreased to 98, 66, 48, 27, 18 and 1 percent following addition of 1, 2.5, 5, 10, 20 and 25 µM ciglitazone, respectively (Fig. 2C). Moreover, NSCs cultured with ATRA also showed a dose-dependent decrease in EGF+bFGF-induced proliferation/viability. While NSCs cultured with EGF+bFGF in the absence of ATRA showed 100% viability that decreased to 98, 65, 44, 42, 22 and 13 percent following addition of 1, 2.5, 5, 10, 20 and 25 µM ATRA, respectively in culture (Fig. 2D). These results suggest that PPARγ agonists inhibit EGF+bFGF-induced proliferation and survival of NSCs in culture.

### PPARγ agonists induce oligodendrocyte differentiation of NSCs

**Figure 3:**
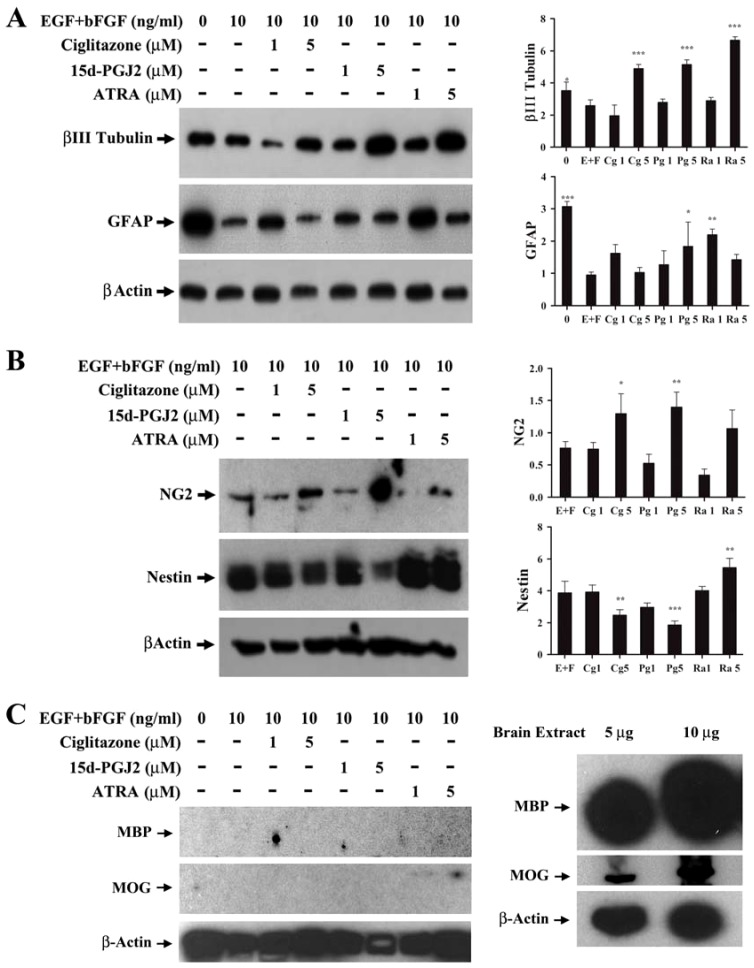
Modulation of stem cell and differentiation markers by PPARγ agonists in NSCs. NSCs were cultured in NBM+B27 with EGF+bFGF in the presence of 0, 1 and 5 µM ciglitazone, 15d-PGJ2 or ATRA at 37°C for 72 h. The expression of βIII tubulin, GFAP (A), NG2, Nestin (B), MBP, MOG (C) and β-Actin was analyzed by Western blot and ECL detection system. Mouse brain extract was used as positive control (C). The relative quantities of protein bands normalized to β-Actin in the blots were determined by densitometry and presented as histograms. The values are mean±SD and the p values are expressed as *(p<0.05), **(p<0.01), and ***(p<0.001). The figure is a representative of five independent experiments.

To study the effect of PPARγ agonists on NSC differentiation we examined the expression of neuro-glial markers by Western blot analysis. As shown in Figure 3A, NSCs cultured in NBM+B27 in the absence of EGF+bFGF expressed elevated levels of neuron-specific β-III tubulin that decreased significantly following addition of 10 ng/ml EGF+bFGF. Interestingly, NSCs cultured with EGF+bFGF in the presence of 5 µM ciglitazone, 15d-PGJ2, or ATRA showed a significant increase in the expression of β-III tubulin compared to EGF+bFGF treated cells. Similarly, NSCs cultured in the absence of EGF+bFGF expressed elevated levels of astrocyte-specific GFAP that decreased significantly following the addition of 10 ng/ml EGF+bFGF. Treatment with 1 or 5 µM ciglitazone, 15d-PGJ2, or ATRA resulted in a partial increase in GFAP expression, reaching statistical significance at 5 µM 15d-PGJ2 and 1 µM ATRA compared to EGF+bFGF control (Fig. 3A). Moreover, NSCs cultured in NBM with EGF+bFGF expressed detectable levels of oligodendrocyte progenitor-specific NG2 proteoglycan that increased significantly after the addition of 5 µM ciglitazone or 15d-PGJ2 but not with ATRA (Fig. 3B). In addition, NSCs cultured with EGF+bFGF showed elevated expression of stem cell marker Nestin that decrease significantly after the addition of 5 µM ciglitazone or 15d-PGJ2 but increased after treatment with ATRA (Fig. 3B). However, NSCs cultured in NBM with EGF+bFGF in the presence of PPARγ agonists failed to express myelin basic protein (MBP) or myelin oligodendrocyte glycoprotein (MOG), as detected in adult mouse brain (Fig. 3C).

**Figure 4:**
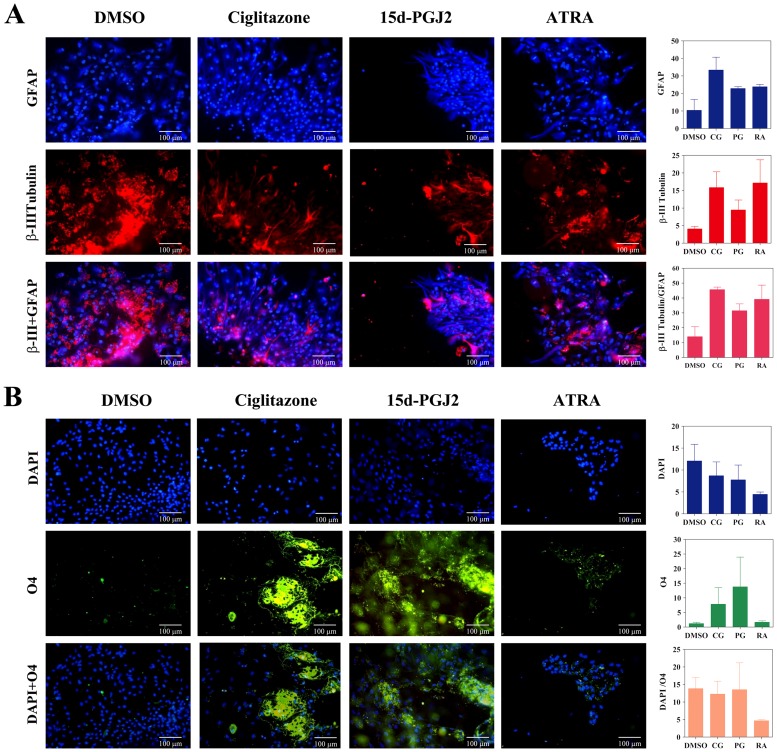
PPARγ agonists induce the expression of oligodendrocyte markers in three days in NSCs. Neurospheres were cultured in poly-D-lysine coated 8 well chamber slides in NBM+B27 with 10 ng/ml EGF+bFGF in the presence of 0 (DMSO) or 1 µM ciglitazone, 15d-PGJ2 or ATRA. After 3 days the cells were stained with GFAP, βIII tubulin, and O4 antibodies along with DAPI and photographed (200×) under fluorescence microscope. The figure is a representative of three independent experiments. The values are mean±SEM and the p values are expressed as *(p<0.05), **(p<0.01), and ***(p<0.001). The figure is a representative of three independent experiments.

**Figure 5:**
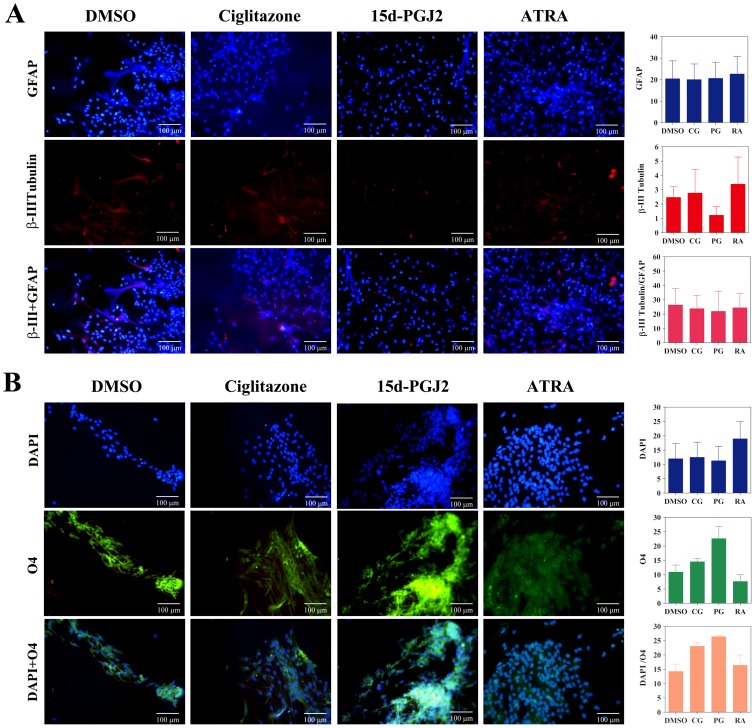
PPARγ agonists induce the expression of oligodendrocyte markers in seven days in NSCs. Neurospheres were cultured in poly-D-lysine coated 8 well chamber slides in NBM+B27 with 10 ng/ml EGF+bFGF in the presence of 0 (DMSO) or 1 µM ciglitazone, 15d-PGJ2 or ATRA. After 7 days the cells were stained with GFAP, βIII tubulin, and O4 antibodies along with DAPI and photographed (200×) under fluorescence microscope. The values are mean±SEM and the p values are expressed as *(p<0.05), **(p<0.01), and ***(p<0.001). The figure is a representative of three independent experiments.

To further determine the effect of PPARγ agonists on NSC differentiation, we examined the expression of neuro-glial markers by immunocytochemical techniques. As shown in Figure 4 and 5, NSCs cultured in NBM+B27 in the presence of EGF+bFGF showed detectable expression of astrocyte-specific GFAP and neuronal β-III tubulin that increased after treatment with ciglitazone, 15d-PGJ2 or ATRA for three and seven days, respectively. Quantitative analysis showed a trend towards increase in GFAP and β-III tubulin expression in NSCs cultured with ciglitazone, 15d-PGJ2 or ATRA compared to DMSO control. In addition, NSCs cultured with EGF+bFGF in the presence of ciglitazone or 15d-PGJ2 showed considerable increase in the expression of pre-oligodendrocyte specific O4 reactivity with characteristic morphology and migration pattern in 3 days that further increased with 15d-PGJ2 by day 7 (Fig. 4, 5). However, NSCs cultured with EGF+bFGF in the absence of PPARγ agonists or in the presence of ATRA showed only minimal O4 reactivity on day 3 with a marginal increase by day 7 (Fig. 4, 5). These findings suggest that PPARγ agonists induce the differentiation of oligodendrocyte progenitor cells from NSCs and may require additional signals to promote their maturation to myelinating oligodendrocytes.

### PPARγ agonists modulate the expression of stemness genes in NSCs

To define the mechanisms by which PPARγ agonists regulate neuro-glial differentiation of NSCs, we analyzed stemness gene profile using TaqMan low density gene array. This array includes a panel of 40 stemness and 50 differentiation genes. As shown in Table 1 and 2, in vitro culture of NSCs in NBM with EGF+bFGF in the presence of PPARγ agonists resulted in significant changes in the expression of many stemness and differentiation genes in three days. Treatment with ciglitazone induced ≥100-fold increase in 22, 1–100 fold increase in 4 and 4 fold decrease in one stemness gene. Moreover, 9 stemness genes expressed in NSCs were undetectable after treatment with ciglitazone (D-ND) and 4 stemness genes were undetected in NSCs cultured in the absence or presence of ciglitazone (ND-ND). Similarly, in vitro treatment of NSCs with 15d-PGJ2 induced ≥100-fold increase in 14, 1–100 fold increase in 5 and ≥100 fold decrease in 2 stemness genes. Moreover, 6 stemness genes expressed in NSCs were undetectable after treatment with 15d-PGJ2 (D-ND), 3 stemness genes undetectable in NSCs were detected after treatment with 15d-PGJ2 (ND-D) and 10 stemness genes remained undetectable following treatment with 15d-PGJ2 (ND-ND). In addition, NSCs treated with ATRA showed ≥100-fold increase in 12, 1–100 fold increase in 5 and 1–1000 fold decrease in 7 stemness genes. Furthermore, 7 stemness genes expressed in NSCs were undetectable after treatment with ATRA (D-ND), 3 stemness genes not detected in NSCs were detected after treatment with ATRA (ND-D), and 5 stemness genes remained undetected after treatment with ATRA (ND-ND) (Table 1). The NSCs cultured with any of the three agonists exhibited elevated expression of 10 stemness factors (Nog, Crabp2, Dnmt3b, Srfp2, Gal, Bxdc2, Podxl, Kit, Lefty1, and Nodal) along with the suppression of 7 other stemness factors (Sox2, Lifr, CD9, Nr6A1, Nanog, Gabrb3 and Ifitm1) (Table 1).

**Table 1:** Regulation of stemness gene profile by PPARγ agonists in neural stem cells.

Symbol	Gene Name	Ciglitazone	15d-PGJ2	Retinoic acid
Nog	Noggin	9.2E18↑	1.6E15↑	4 E12↑
Crabp2	Cellular retinoic acid binding protein 2	9.2E18↑	ND-D↑	4.8E14↑
Commd3	COMM domain containing 3	9.2E18↑	ND-ND↓	8.1E12↑
Dnmt3b	DNA cytosine-5-methyltransferase 3b	1.4E17↑	1.8E12↑	1.6E12↑
Srfp2	Frizzled-related protein	7.1E13↑	2.5E7↑	4082↑
Gdf3	Growth differentiation factor 3	1.9E12↑	5771↑	ND-ND↓
Rest	RE1-silencing transcription factor	3.3E11↑	ND-ND↓	0.001↓
Igfbp2	Insulin-like growth factor binding protein 2	1.3E11↑	ND-ND↓	1.7↑
Nes	Nestin	3.0E10↑	ND-ND↓	1439↑
Gal	Galanin prepropeptide	5.8E8↑	1.2E9↑	24.5↑
Pou5f1	POU class 5 homeobox 1	2.3E8↑	3.8E14↑	0.022↓
Xist	X (inactive)-specific transcript	8.05E7↑	9.17E9↑	ND-ND↓
Zfp42	Zinc finger protein 42 homolog	8.8E6↑	18.8↑	ND-ND↓
Bxdc2	BRX1, biogenesis of ribosomes, homolog	1.1E6↑	7.6E9↑	9.5E6↑
Podxl	Podocalyxin-like	2.8E5↑	7.8E14↑	16.1↑
Tdgf1	Teratocarcinoma-derived growth factor 1	4.3E4↑	14.7↑	0.003↓
Lin 28	Lin-28 homolog A	2.1E4↑	ND-ND↓	D-ND↓
Fgf5	Fibroblast growth factor 5	1.8E4↑	D-ND↓	0.11↓
Utf1	Undifferentiated embryonic cell transcription factor	1.4E4↑	2.9↑	D-ND↓
Pten	Phosphatase and tensin homolog	6799↑	ND-ND↓	ND-ND↓
Kit	Feline sarcoma viral oncogene	757.2↑	191.9↑	8.1E11↑
Lefty1	Left-right determination factor 1	112.4↑	5.9E11↑	7.2E11↑
NR5a2	Nuclear receptor subfamily 5	12.1↑	0.003↓	ND-D↑
Ifitm2	Interferon induced transmembrane protein 2	11.8↑	1.4E7↑	D-ND↓
Nodal	Nodal homolog	9.8↑	0.002↓	1.5E10↑
Lefty2	Left-right determination factor 2	1.2↑	ND-D↑	1.6↑
Grb7	Growth factor receptor-bound protein 7	0.4↓	ND-D↑	2.5E7↑
Fgf4	Fibroblast growth factor 4	D-ND↓	4.3E16↑	2.6E14↑
Gbx2	Gastrulation brain homeobox 2	D-ND↓	6.3E10↑	2.5↑
Tert	Telomerase reverse transcriptase	D-ND↓	16.4↑	0.002↓
Tfcp2l1	Transcription factor CP2-like 1	D-ND↓	8.9↑	0.003↓
Sox2	SRY-box 2	D-ND↓	D-ND↓	0.011↓
Lifr	Leukemia inhibitory factor receptor alpha	D-ND↓	D-ND↓	D-ND↓
CD9	CD9 molecule	D-ND↓	D-ND↓	D-ND↓
Nr6a1	Nuclear receptor subfamily 6, group A, member 1	D-ND↓	D-ND↓	D-ND↓
Nanog	Nanog homeobox	D-ND↓	ND-ND↓	D-ND↓
Gabrb3	GABA A receptor, beta 3	ND-ND↓	D-ND↓	0.5↓
Il6st	Interleukin 6 signal transducer	ND-ND↓	ND-ND↓	ND-D↑
Sema3a	Sema domain, immunoglobulin domain (Ig)	ND-ND↓	ND-ND↓	ND-D↑
Ifitm1	Interferon induced transmembrane protein 1	ND-ND↓	ND-ND↓	ND-ND↓

NSCs were cultured in NBM with EGF+bFGF in the presence of DMSO, ciglitazone, 15d-PGJ2 or ATRA for 3 days. The stemness gene expression was analyzed by qRT-PCR using a 384 gene card array. The fold change was calculated using automatic threshold setting and is based on expression levels in DMSO treated cells as 1 after normalizing to 18S or GAPDH. The genes in the table are arranged from high to low expression in ciglitazone treated cells. ND, not detected and D, detected. Arrows indicate up or down regulated genes. This data is a representative of two independent experiments.

**Table 2:** Regulation of differentiation gene profile by PPARγ agonists in neural stem cells.

Symbol	Gene Name	Ciglitazone	15d-PGJ2	Retinoic acid
Col1a1	Collagen, type I, alpha 1	ND-D↑	0.6↓	1.1↑
Ptf1a	Pancreas specific transcription factor 1a	ND-D↑	ND-ND↓	1.9E14↑
Col2a1	Collagen, type II, alpha 1	ND-D↑	ND-ND↓	ND-D↑
Pecam 1	Platelet/endothelial cell adhesion molecule	ND-D↑	3.3E14↑	5.7E10↑
Hbb	Hemoglobin, beta	ND-D↑	443.2↑	2.2E13↑
Foxd3	Forkhead box D3	ND-D↑	ND-ND↓	ND-ND↓
Pax6	Paired box 6	9.2E18↑	6.3E4↑	3.4E10↑
Sst	Somatostatin	2.5E18↑	1.1E15↑	3628↑
Gcg	Glucagon	1.6E18↑	1.4E15↑	7.4E13↑
Lamb1	Laminin, beta 1	3.8E16↑	4.8E14↑	D-ND↓
Krt1	Keratin 1	5.3E15↑	ND-D↑	1.5E11↑
Des	Desmin	9.6E14↑	7.0↑	0.002↓
Cd34	CD34 molecule	3.6E12↑	ND-ND↓	ND-ND↓
Lama1	Laminin, alpha 1	2.8E12↑	0.002↓	948.2↑
Neurod1	Neurogenic differentiation 1	3.8E11↑	4.8E13↑	5.0E10↑
Gfap	Glial fibrillary acidic protein	2.9E11↑	ND-D↑	0.2↓
Hlxb9	Motor neuron and pancreas homeobox 1	8.8E10↑	1.3E9↑	1.1E5↑
Wt1	Wilms tumor 1	2.5E10↑	0.001↓	0.022↓
Flt1	Fms-related tyrosine kinase 1	1.2E10↑	143.4↑	1.6E8↑
Eras	ES cell expressed Ras	4.7E7↑	4.6E14↑	3.2E11↑
T	T, brachyury homolog	1.2E7↑	5.3E14↑	8.0E13↑
Fn1	Fibronectin 1	1.7E6↑	159.3↑	D-ND↓
Pax4	Paired box 4	1.3E6↑	3.7↑	0.005↓
Sycp3	Synaptonemal complex protein 3	6.1E5↑	2.1E8↑	ND-ND↓
Th	Tyrosine hydroxylase	8.7E5↑	0.3↓	0.003↓
Eomes	Eomesodermin	3.9E4↑	ND-ND↓	1.1E7↑
Actc1	Actin, alpha, cardiac muscle 1	12335↑	14.6↑	55.8↑
Tat	Tyrosine aminotransferase	58.9↑	2052↑	4.6E9↑
Iapp	Islet amyloid polypeptide	47.2↑	0.5↓	0.001↓
Serpina1	Serpin peptidase inhibitor, clade A member 1	33.2↑	0.579↓	6.248↑
Olig2	Oligodendrocyte transcription factor 2	20.1↑	6.3E11↑	8.2E9↑
Nppa	Natriuretic peptide A	19.7↑	0.005↓	ND-ND↓
Ins2	Insulin	15.9↑	D-ND↓	D-ND↓
Ipf1	Pancreatic and duodenal homeobox 1	0.1↓	1.1E4↑	4.8E8↑
Gata 6	GATA binding protein 6	0.051↓	0.008↓	D-ND↓
Runx2	Runt-related transcription factor 2	D-ND↓	1.3E15↑	7.5E4↑
Cdh5	Cadherin 5, type 2	D-ND↓	1.4E9↑	6.3E14↑
Sox17	SRY-box 17	D-ND↓	5182↑	ND-ND↓
Myod1	Myogenic differentiation 1	D-ND↓	28.58↑	D-ND↓
Ddx4	DEAD (Asp-Glu-Ala-Asp) box polypeptide 4	D-ND↓	ND-D↑	ND-ND↓
Isl1	ISL LIM homeobox 1	D-ND↓	0.043↓	D-ND↓
Afp	Alphafeto protein	D-ND↓	202.5↑	94↑
Hbz	Hemoglobin, zeta	D-ND↓	D-ND↓	3.2E5↑
Myf5	Myogenic factor 5	ND-ND↓	7.27E12↑	3.86E12↑
Foxa2	Forkhead box A2	ND-ND↓	D-ND↓	D-ND↓
Gcm1	Glial cells missing homolog 1	ND-ND↓	ND-ND↓	ND-ND↓
Gata4	GATA binding protein 4	ND-ND↓	ND-ND↓	ND-ND↓
Lamc 1	Laminin, gamma 1	ND-ND↓	ND-ND↓	ND-D↑
Syp	Synaptophysin	ND-ND↓	ND-ND↓	ND-D↑
Cdx2	Caudal type homeobox 2	ND-ND↓	ND-D↑	ND-ND↓

NSCs were cultured in NBM with EGF+bFGF in the presence of DMSO, ciglitazone, 15d-PGJ2 or ATRA for 3 days. The differentiation gene expression profile was analyzed by qRT-PCR using a 384 gene card array. The fold change was calculated using automatic threshold setting and is based on expression levels in DMSO treated cells as 1 after normalizing to 18S or GAPDH. The genes are arranged in the table from high to low expression in ciglitazone treated cells. ND, not detected and D, detected. Arrows indicate up or down regulated genes. This data is a representative of two independent experiments.

**Figure 6:**
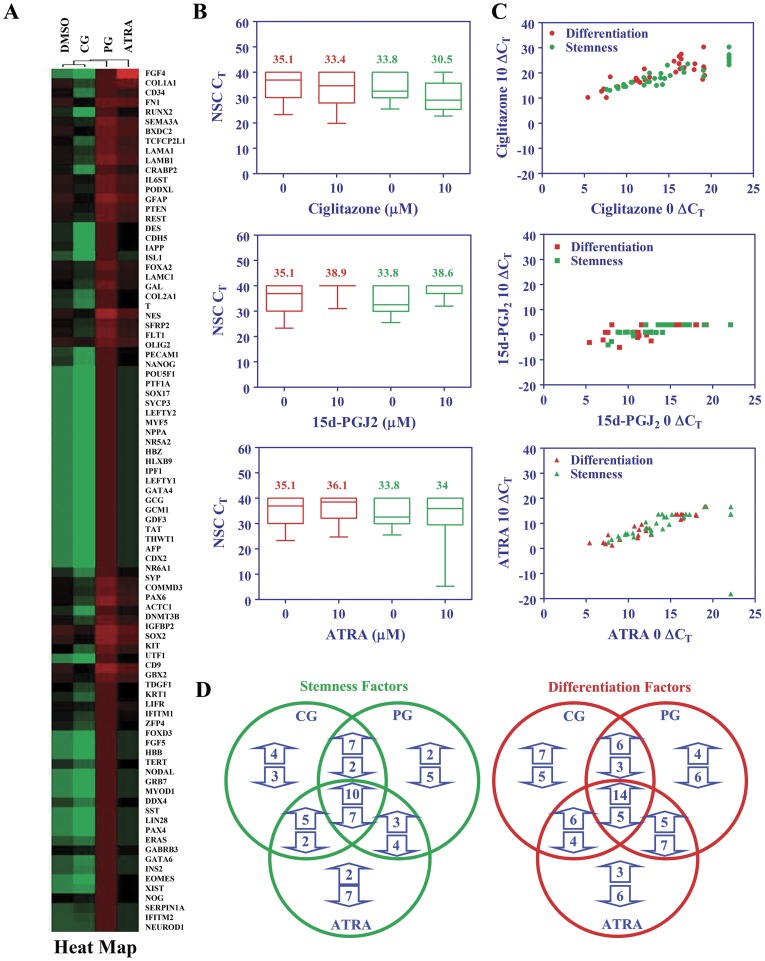
Regulation of stemness and differentiation genes by PPARγ agonists in NSCs. NSCs were cultured in NBM+B27 with 10 ng/ml EGF+bFGF in the presence of 0 or 1 µM ciglitazone, 15d-PGJ2 or ATRA for 3 days and the stem cell gene expression analyzed by qRT-PCR. (A) Heat map showing the expression levels of stemness and differentiation genes in NSCs treated with agonists compared to control. (B) Box plots showing the C_T_ values of differentiation (Red) and stemness (Green) genes in NSCs treated with agonists compared to control. (C) Scatter plots showing ΔC_T_ values of differentiation (Red) and stemness (Green) genes in NSCs treated with agonists compared to control. (D) Number of stemness and differentiation genes altered is presented as Venn diagram. The figure is representative of two independent experiments.

Further analyses revealed significant alterations in the stemness gene expression profile of NSCs that were common or distinct among treatment groups. As shown in Figure 6A, the heat map demonstrates altered expression of many stemness genes following treatment of NSCs with PPARγ agonists when compared to controls. The C_T_ values presented as box plots (Fig 6B) also demonstrates changes in the expression of stemness genes in NSCs by PPARγ agonists. The horizontal line represents the median while the numbers represent the mean. We found that ciglitazone induced a mild suppression of stemness genes, while 15d-PGJ_2_ increased overall expression of stemness genes. Treatment with ATRA showed no marked difference in the expression of stemness genes. Scatter plots of ΔC_T_ values (Fig. 6C) further confirmed the altered expression of stemness genes in NSCs following treatment with PPARγ agonists in culture. Moreover, as shown in Figure 6D, Venn diagram demonstrates elevated expression of 10 stemness genes by all three agonists, 7 by ciglitazone and 15d-PGJ2, 3 by 15d-PGJ2 and ATRA and 5 by ciglitazone and ATRA in NSCs. We have also found a decrease in the expression of 7 stemness genes by all three agonists, 2 by ciglitazone and 15d-PGJ2, 4 by 15d-PGJ2 and ATRA and 2 by ciglitazone and ATRA in NSCs. While 4 stemness genes were elevated only by ciglitazone, 2 by 15d-PGJ2 and 2 by ATRA, 3 stemness genes were inhibited only by ciglitazone, 5 by 15d-PGJ2 and 7 by ATRA in NSCs (Fig. 6D). These findings suggest that PPARγ agonists regulate self-renewal and differentiation by modulating stemness gene expression profile in NSCs.

### PPARγ agonists modulate the expression of differentiation genes in NSCs

We then examined the expression of 50 differentiation factors in NSCs cultured with PPARγ agonists. As shown in Table 2, NSCs cultured with ciglitazone showed ≥100-fold increase in 21, 1–100 fold increase in 6, and 1–100 fold decrease in 2 differentiation genes. Meanwhile, 6 differentiation genes not expressed in NSCs were detected after treatment with ciglitazone (ND-D), 8 differentiation genes expressed in NSCs were not detected after treatment with ciglitazone (D-ND) and 7 differentiation genes were not detected (ND-ND) in NSCs cultured in the absence or presence of ciglitazone. Similarly, in vitro treatment of NSCs with 15d-PGJ2 induced ≥100 fold increase in 21, 1–100 fold increase in 4 and 1–1000 fold decrease in 8 differentiation genes. Moreover, 3 differentiation genes expressed in NSCs were undetectable after treatment with 15d-PGJ2 (D-ND), 4 differentiation genes not detected in NSCs were detected after treatment with 15d-PGJ2 (ND-D) and 9 differentiation genes remained undetected in NSCs cultured in the absence or presence of 15d-PGJ2 (ND-ND). In addition, in vitro treatment of NSCs with ATRA induced ≥100-fold increase in 21, 1–100 fold increase in 4 and 1–1000 fold decrease in 6 differentiation genes. Moreover, 7 differentiation genes expressed in NSCs were undetectable after treatment with ATRA (D-ND), 3 differentiation genes not detected in NSCs were detected after treatment with ATRA (ND-D) and 9 differentiation genes remained undetected in NSCs after treatment with ATRA (ND-ND) (Table 2). Among the differentiation genes altered by ciglitazone or 15d-PGJ2 in NSCs, the expression of 6 differentiation factors increased, whereas the expression of 3 differentiation factors decreased (Table 2). NSCs treated with any of the three drugs in this study exhibited elevated expression of 14 differentiation factors (Pecam1, Hbb, Pax6, Sst, Gcg, Krt1, Neurod1, Hlxb9, Flt1, Eras, T, Actc1, Tat, and Olig2) with the suppression of 5 differentiation factors (Gata6, Isl1, Foxa2, Gcm1 and Gata4) (Table 2).

We have also found significant alterations in the differentiation gene expression profile of NSCs that were common or distinct among treatments. As shown in Figure 6A, heat map demonstrates altered expression of many differentiation genes in NSCs following treatment with PPARγ agonists when compared to controls. The C_T_ values presented as box plots (Fig 6B) demonstrates changes in the expression of differentiation genes induced by PPARγ agonists in NSCs. We found that ciglitazone induced a mild suppression of differentiation genes, while 15d-PGJ_2_ increased the overall expression of differentiation genes, while ATRA showed no effect. Scatter plots of ΔC_T_ values (Fig. 6C) further confirmed the altered expression of differentiation genes by PPARγ agonists in NSCs. Moreover, as shown in Figure 6D, Venn diagram demonstrates an elevated expression of 14 differentiation genes by all three agonists, 6 by ciglitazone and 15d-PGJ2, 5 by 15d-PGJ2 and ATRA and 6 by ciglitazone and ATRA in NSCs. We also found a decrease in the expression of 5 differentiation genes by all three agonists, 3 by ciglitazone and 15d-PGJ2, 7 by 15d-PGJ2 and ATRA and 4 by ciglitazone and ATRA in NSCs. While 7 differentiation genes were elevated only by ciglitazone, 4 by 15d-PGJ2 and 3 by ATRA, 5 differentiation genes were inhibited only by ciglitazone, 6 by 15d-PGJ2 and 6 by ATRA in NSCs (Fig. 6D). These results suggest that PPARγ agonists promote neuro-glial differentiation by modulating distinct stemness and differentiation gene expression profile in NSCs.

## Discussion

The past decade has seen tremendous progress in understanding the molecular mechanisms in the regulation of growth, self-renewal and differentiation of stem cells along specific lineages. This progress was made possible by the discovery of myriad growth factors including EGF and bFGF and their signaling pathways responsible for maintaining self-renewal and pluripotency of stem cells in culture. PPARγ is an important regulator of growth and differentiation of many cell types during pre and post natal development. In this study we found that NSCs cultured in NBM+B27 with EGF+bFGF grow and expand as neurospheres, but PPARγ agonists decrease growth and enhance neuro-glial differentiation in culture. Among the many neuro-glial markers analyzed, we observed a significant increase in the expression of NG2 in PPARγ agonist treated NSCs, suggesting oligodendrocyte progenitor differentiation of NSCs in culture. Moreover, an increase in the expression of antigens detected by O4 antibody, suggests pre-oligodendrocyte differentiation of NSCs following treatment with PPARγ agonists. These findings are consistent with previous reports showing the detection of NG2 and O4 as markers of NSC differentiation to oligodendrocyte progenitor cells in culture. Stabenfeldt et al., used O4 as a marker for oligodendrocyte differentiation of mouse NSCs in 7 day old cultures [43]. A study by Sypecka et al., using human cord blood derived NSCs also demonstrated oligodendroglial differentiation by O4 staining [44]. Sher et al., used several markers to identify various stages of oligodendrocyte differentiation (PDGFRα - precursor, progenitor, NG2 - progenitor, RIP - preoligodendrocyte and MBP - myelinating mature oligodendrocyte) in NSCs derived from C57BL/6 mice [45]. However, we found that NSCs cultured in the presence of PPARγ agonists failed to express MBP or MOG as detected in mouse brain extracts, suggesting that additional signals are required to induce maturation of oligodendrocytes.

Growth and differentiation signals are integrated by key transcription factors which regulate specific gene clusters to allow proliferation or differentiation to acquire specialized functions in NSCs. To define the mechanisms by which PPARγ agonists regulate growth and self-renewal of NSCs, we analyzed the expression of 40 stemness genes. Among the many altered stemness genes, Sox2 was one of the important genes inhibited by PPARγ agonists and ATRA in NSCs. Sox2 is a key member of the Sox (SRY-like HMG box) family transcription factors expressed in embryonic stem cells (ES), NSCs and trophoblast stem cells, but not in differentiated cells and is essential for maintaining pluripotency [46]–[49]. CD9 is a surface protein expressed in neural progenitor cells [50] that was also suppressed by PPARγ agonists. The suppression of Sox2, CD9 and other stemness factors in NSCs suggests the inhibition of self-renewal and stemness by PPARγagonists. We have also observed the upregulation of many stemness genes by PPARγ agonists in NSCs. Among them Noggin, a stemness gene implicated in neurogenesis and the formation of anterior neural patterning [51], was elevated by PPARγ agonists and ATRA. PPARγ agonists and ATRA also increased the expression of cellular retinoic acid binding protein (Crabp2), known to mediate retinoic acid induced motor neuron differentiation [52]. Moreover, PPARγ agonists and ATRA increased the expression of Galanin, a protein essential for the development and survival of a subset of dorsal root ganglia cells [53] and basal forebrain cholinergic neurons [54]. This is consistent with earlier studies showing the upregulation of Galanin mRNA and protein levels after sciatic [55], facial [56] or vagal [57] nerve injury, suggesting its involvement in nerve repair. Our findings suggest that the down-regulation of a subset of stemness genes is sufficient to inhibit growth and self-renewal of NSCs.

To further determine the mechanism in the promotion of neuro-glial differentiation of NSCs by PPARγ agonists, we analyzed the expression of 50 differentiation genes. Oligodendrocyte differentiation factor 2 (Olig2) is one of the many differentiation factors elevated following treatment with PPARγ agonists that is critical in maintaining oligodendrocyte phenotype [58]. Therefore, elevated Olig2 and O4 expression as demonstrated in this study could be a mechanism by which PPARγ agonists promote oligodendrocyte differentiation of NSCs. We have also found that PPARγagonists increase the expression of glial fibrillary acidic protein (GFAP) in NSCs, indicating astrocyte differentiation in culture. PPARγ agonists and ATRA also induced the expression of Pecam1 in NSCs. Earlier studies have shown that PPARγ ligands induce gastro-protective and ulcer healing properties by increasing the expression of Pecam-1 [59]. Pecam-1 expressed in NSCs residing in specialized niches closely associated with blood vessels in adult brain [60], [61] may mediate cross-talk with endothelial cells (ECs) to regulate neurogenesis and angiogenesis [62]. PPARγ agonists and ATRA also induced the expression of Neurogenic differentiation factor (Neurod1), a member of the basic helix-loop-helix (bHLH) transcription factor that plays a role in the development of nervous and endocrine systems [63]. Neurod1-null mice exhibit behavioral abnormalities due to a reduction in sensory neurons and Neurod1 regulates insulin gene expression by binding to a critical E-box motif on insulin promoter [64]. Pancreas specific transcription factor 1a (Ptf1a), involved in the maturation of pancreatic β cells, insulin production and glucose homeostasis [65], [66] is also induced by ciglitazone and ATRA in NSCs. Alphafeto protein (Afp) expression was enhanced in NSCs following treatment with 15d-PGJ2 or ATRA. AFP is expressed in early embryos, hematopoietic progenitor cells and in adult brain [67] and PPARγ agonists upregulate Afp expression and differentiation of hepatic oval cells [68]. Thus the upregulation of selective differentiation factors could be a mechanism by which PPARγagonists promote neuro-glial differentiation of NSCs. Other studies have demonstrated that in vitro differentiation and maturation of oligodendrocytes depends on many factors, including T3 function, ECM interactions and modulation of signaling pathways [69], [70]. We have also found an increase in the expression of Olig2 and other genes relevant to oligodendrocyte differentiation of NSCs following exposure to PPARγ agonists. Our future studies will further explore the role of specific stemness and differentiation genes altered by PPARγ agonists in promoting neuro-glial differentiation of NSCs. Our findings suggest that PPARγ agonists could prove beneficial in the treatment of neurodegenerative diseases.

Earlier studies have reported conflicting results on the influence of PPARγ agonists on NSCs. Wada et al., reported elevated expression of PPARγ in NSCs and PPARγagonists induce proliferation and inhibited neuronal differentiation by activating EGFR/ERK pathway, which are attenuated in PPARγ^+/−^ and PPARγ-silenced NSCs in culture [40]. Morales-Garcia et al., showed that PPARγ agonists increase the number of proliferating NSCs in the subventricular zone and rostral migration in adult rats and neurosphere formation and differentiation of NSCs that are blocked by PPARγ antagonists in culture [41]. On the other hand, Katura et al., reported a novel biphasic effect of 15d-PGJ2 on EGF-induced proliferation of NSCs with an increase at lower doses (≤0.3 µM) and suppression at higher doses (0.5–10 µM) in culture [42]. In this study we demonstrated that PPARγ agonists inhibit EGF+bFGF-induced proliferation of NSCs at 1 to 25 µM doses in culture. We have also found that PPARγ agonists promote neuro-glial differentiation by modulating stemness and differentiation genes in NSCs. We believe that the discrepancy between earlier reports and our results on the effect of PPARγ agonists on NSCs could be due to difference in culture conditions used. In particular, we performed all our proliferation and differentiation assays by culturing NSCs in NBM+B27 with EGF+bFGF in the absence or presence of PPARγ agonists, while in other studies NSCs were cultured with PPARγ agonists in the absence of EGF+bFGF [40]. Earlier studies have used serum containing medium in the absence of EGF and bFGF that promote spontaneous differentiation of NSCs into different types of neuro-glial cells in culture. However, our objective was to promote selective lineage specific differentiation of NSCs. We have shown earlier that PPARγ agonists regulate leukemia inhibitory factor (LIF) induced growth and self-renewal of mouse embryonic stem cells by modulating Jak-Stat signaling pathway [71], [72]. We reasoned that PPARγ agonists would regulate selected signaling pathways induced by EGF+bFGF in stem cell culture condition. We also believe that the discrepancy between earlier reports and our results could be due to difference in the PPARγ agonists and the dose-ranges used. In particular, in earlier studies the growth inducing effect on NSCs was observed only at lower doses of PPARγ agonists, while higher doses caused an anti-proliferative effect, which is consistent with our findings [40]. Thus further studies on the regulation of NSCs by PPARγ agonists would help to determine their use in the treatment of neurodegenerative diseases.

## Materials and Methods

### Reagents

The murine recombinant epidermal growth factor (EGF) was purchased from PeproTech (Rocky Hill, NJ) and basic fibroblast growth factor (bFGF) was purchased from R&D Systems (Minneapolis, MN). Ciglitazone was purchased from CalBiochem (La Jolla, CA), while 15-Deoxy-Δ^12,14^-Prostaglandin J_2_ (15d-PGJ2) came from Sigma Chemicals (St Louis, MO). Anti-β-Actin antibody was purchased from Santa Cruz Biotechnology Inc. (Santa Cruz, CA). The HRP conjugated secondary Abs, all-trans retinoic acid (ATRA) and other chemicals were purchased from Sigma Chemicals Co. (St Louis, MO). WST-1 reagent (4-[3-(4-iodophenyl)-2-(4-nitrophenyl)-2H-5-tetrazolio]-1,3-benzene disulfonate) was purchased from Roche (Indianapolis, IN). Primary antibodies specific to glial fibrillary acidic protein (GFAP, anti-goat polyclonal IgG, sc-6170), beta III tubulin (βIII tubulin, anti-mouse mAb IgG, sc-51670), Nestin (goat polyclonal IgG, sc-21248), myelin basic protein (MBP, anti-goat polyclonal IgG, sc-13912), myelin oligodendrocyte glycoprotein (MOG, anti-mouse mAb IgG, sc-376138), Neural/Glial Antigen 2 (NG2, anti-rabbit polyclonal IgG, sc-20162) and horse radish peroxidase (HRP) conjugated secondary antibodies (Goat anti-rabbit IgG, sc-2004; donkey anti-goat IgG, sc-2020; goat anti-mouse IgG, sc-2031) were purchased from Santa Cruz Biotechnology Inc. (Santa Cruz, CA). The mouse monoclonal oligodendrocyte progenitor marker O4 antibody (Clone 81-IgM); GFAP (rabbit polyclonal IgG, 01415) and fluorochrome conjugated secondary antibodies (AMCA conjugated goat anti-rabbit polyclonal IgG, 10214; Texas red conjugated goat anti-mouse mAb IgG, 10213; FITC conjugated goat anti-mouse IgM, 10211) were purchased from Stem Cell Technologies (Vancouver, Canada). The 384 well TaqMan low density mouse stem cell array and other PCR reagents were obtained from Applied Biosystems (Foster City, CA).

### Cell culture

C57BL/6 mice were obtained from Harlan (Indianapolis, IN) and the breading colonies were maintained in the animal care facility at Methodist Research Institute. All animal protocols used in the experiments were approved by the Institutional Animal Care and Use Committee. Primary mouse NSCs were generated by culturing dissociated brain cells from new born (post natal 0–3 day) C57B/6 mice in neurobasal medium (NBM) supplemented with B27 in the presence of 10 ng/ml bFGF and EGF. The cells were cultured in 12 well tissue culture plates in 5% CO_2_ incubator at 37°C with a medium change on every 2–3 days. The neurospheres generated in 7–10 days were photographed under phase contrast microscope (AX70, Olympus Optical, Japan).

### Proliferation assay

Proliferation of NSCs was measured by ^3^H thymidine uptake and WST-1 assays. Briefly, NSCs obtained by dissociating neurospheres using accutase (Invitrogen) were cultured in 96-well tissue culture plates (1×10^4^/200 µl/well) in NBM with B27 in the absence or presence of 10 ng/ml EGF, bFGF or EGF+bFGF. ^3^H thymidine (0.5 µCi/well) was added at 48 h and the cells were harvested after 72 h using a Tomtech harvester 96 (Hamden, CT, USA). The amount of ^3^H thymidine uptake was counted on Wallac Microbeta liquid scintillation counter (Perkin Elmer, Fremont, CA) as a measure of proliferation. For WST-1 assay, NSCs were cultured in 96-well tissue culture plates (1×10^4^/200 µl/well) in NBM with B27 and 10 ng/ml EGF+bFGF in the presence of 0, 1, 2.5, 5, 10, 20 and 25 µM ciglitazone, 15d-PGJ2 or ATRA. After 72 h, 10 µl of WST-1 reagent was added to each well and the absorbance determined at 450 nm using a titer-plate reader (Alpha Diagnostics, San Antonio, TX).

### Immunocytochemistry

Neurospheres generated by culturing brain cells from newborn mice were transferred to poly-D-lysine coated 8 well chamber slides (BD Biosciences, San Jose, CA) with NBM+B27 and 10 ng/ml of EGF+bFGF. We have added DMSO vehicle in the absence or presence of 1.0 µM ciglitazone, 15d-PGJ2 or ATRA in two identical wells. After 3 and 7 days the cells were fixed with 1% paraformaldehyde in PBS for 15 min and stained with a combination of primary and secondary antibodies by indirect immunofluorescence technique. The cells in one set were stained with rabbit polyclonal IgG specific to GFAP followed by AMCA conjugated polyclonal anti-rabbit IgG and mouse monoclonal IgG specific to βIII-tubulin followed by Texas red conjugated mouse monoclonal IgG (Fig 4A and Fig 5A). Other set of cells were stained with mouse monoclonal IgM (O4) followed by FITC conjugated anti-mouse IgM and DAPI (Fig 4B and Fig 5B). The images were photographed using Leica Leitz DMRB fluorescent microscope (Leica Microsystems, Buffalo Grove, IL, USA) and presented as individual or merged pictures. Quantitative analysis of immunofluorescence was performed using ImageJ software (NIH, http://rsbweb.nih.gov/ij/) and presented as histograms.

### Western Blot Analysis

To examine the effect of PPARγ agonists on neuro-glial differentiation, NSCs were cultured in NBM+B27 with 10 ng/ml EGF+bFGF in the presence of 0, 1, and 5 µM ciglitazone, 15d-PGJ2 or ATRA at 37°C. The cells were harvested after 72 h and whole cell lysates prepared using lysis buffer (0.2 M Tris-HCl pH 6.8, 0.8% SDS, 4% Glycerol, 0.588 M β-mercaptoethanol, 0.05 M EDTA, 8 µg/ml bromophenol blue) for 5 min. Adult C57BL/6 mouse brain was homogenized in lysis buffer and used as positive control. The total protein samples were resolved on 8% (Nestin and NG-2) or 12% (β-III Tubulin, GFAP, MBP and MOG) SDS-PAGE, transferred to nylon (PVD) membrane (BioRad, Hercules, CA), and the residual binding sites blocked by incubation with TBST (10 mM Tris-HCl, pH 8.0, 150 mM NaCl, and 0.05% Tween 20) containing 3% BSA for 1 h. Membranes were incubated with anti-βIII tubulin, anti-GFAP, anti-NG2, anti-Nestin, anti-MBP, anti-MOG or anti-β-Actin antibody (1∶200–500) in TBST containing 1% BSA at 4°C overnight. The blots were washed and incubated with horseradish peroxidase (HRP)-conjugated secondary antibodies in TBST (1∶2500–5000) for 1 h and developed using enhanced chemiluminescence (ECL) detection system and film (Amersham Life Science, Arlington Heights, IL) according to manufacturer's instructions. Quantitative analyses of Western blots were performed using FluorChem HD2 software (Alpha Innotech/Quansys Biosciences, West Logan, Utah).

### Quantitative reverse transcription polymerase chain reaction

To determine the effect of PPARγ agonists on the expression of stemness and differentiation genes, NSCs were cultured in NBM+B27 with 10 ng/ml EGF+bFGF in the presence of 0 or 1 µM ciglitazone, 15d-PGJ2 or ATRA at 37°C for 3 days. Total RNA was extracted using TRIzol reagent (Invitrogen, Carlsbad, CA) and equal amount of RNA was then reverse transcribed into cDNA using TaqMan reverse transcription kit (Applied Biosystems, Foster City, CA). Quantitative reverse transcription polymerase chain reaction (qRT-PCR) was performed using 384-well TaqMan Low Density Mouse Stem Cell Gene Array Card with 90 primer sets in 7900 HT fast Real time PCR system (Applied Biosystems, Foster City, CA). The data were analyzed using the ABI Prism 7900 relative quantification (delta-delta-Ct) study software (Applied Biosystems, Foster City, CA) and the gene expression levels were normalized to 18S or GAPDH and presented as relative fold change (RQ) compared to control. Heat map was constructed using the DataAssist software (Applied Biosystems, Foster City, CA, USA). Box plot, scatter plot, and Venn diagram were generated using GraphPad Prizm 5.0 software (GraphPad, La Jolla, CA, USA).

### Statistical analysis

The experiments were repeated three or more times and the values are expressed as mean±SD/SEM. The differences between groups were analyzed by one way ANOVA using GraphPad Prism 5.0 software and the values * (p<0.05), ** (p<0.01), and *** (p<0.001) were considered significant.
